# Editorial: Marine invertebrates: neurons, glia, and neurotransmitters

**DOI:** 10.3389/fncir.2023.1327991

**Published:** 2023-11-14

**Authors:** Tatiana N. Olivares-Bañuelos, Arturo Ortega

**Affiliations:** ^1^Instituto de Investigaciones Oceanológicas, Universidad Autónoma de Baja California, Ensenada, Mexico; ^2^Department of Toxicology, Centro de Investigaciòn y de Estudios Avanzados del Instituto Politécnico Nacional, Mexico City, Mexico

**Keywords:** neurogenesis, neurodevelopment, sea-animal models, neuroevolution, neurotransmission

The neurosciences are an important area of research that has implications for the transduction of cellular signals in physiology, pharmaceutical, psychological, evolutionary, and medical sciences. Having animal models that allow ethical and scientific research in all these areas represents an important challenge. For decades, it has been shown that invertebrates, particularly aquatic species, are suitable models to analyze the processes of neurotransmission, neurogenesis, neurodevelopment neuronal plasticity, neurodegeneration, neurotoxicity, and even neuroevolutionary processes.

Marine invertebrates have a simple and well-organized nervous system structured by few, accessible, and large nerve cells. The close phylogenetic relationship between Chordata and invertebrate phyla like the Echinodermata allows us to understand the functionality, physiology, plasticity, signaling, differentiation, and neurogenesis between the cells that make up the nervous system. These characteristics make marine invertebrates important model systems for the neurosciences. The results generated with these models allow us to understand the complexity of the nervous system since this information can be extrapolated to the mammalian SN to develop novel molecular alternatives for the treatment of neurodegenerative diseases.

The aim of the present Research Topic is to have an updated compilation of marine invertebrate neuroscience which includes neuron and glial cellular morphology and ultrastructure, signaling, transport mechanisms, neogenesis, differentiation, and cellular trans-differentiation. Manuscripts include a review and original research works.

Mashanov et al. provide us with a complete and well-documented article about the organization and function of the echinoderm radial glia cells, with the primary focus on the role of these cells in adult neurogenesis. Echinoderms are interesting marine deuterostomes with the capacity to regenerate their Central Nervous System (CNS). This remarkable characteristic is important due to their taxa proximity with chordates and hemichordates (Holland, 2020), in which the CNS is more complex in terms of architecture and functionality. Interestingly, adult echinoderms have radial glia cells (until now the only known glial cell type), which are directly involved in (1) neurogenesis processes after either injury or autotomy, (2) nervous system secretory activity, (3) promoting phagocytosis of dying or damaged cells, and (4) forming the support scaffold of the neuroepithelium. In the adult mammalian CNS, radial glia cells are present only in the retina, cerebellum, and olfactory bulb since during embryogenesis, most of these cells are used as progenitors for neuronal and glia cells. In contrast, in other adult species of chordates, like the zebrafish, radial glia cells are abundant (Fujita et al., 2020; Jurisch-Yaksi et al., 2020; Zhang et al., 2021). This review highlights the role of radial glia cells in adult neurogenesis in order to understand the extraordinary plasticity of these echinoderm cells.

Kosevich shows us evidence of the existence of hydroids' colonial CNS. Hydroids belong to phylum Cnidaria, and Bilateria and Cnidaria clades are phylogenetically close which makes the first ones an interesting group of organisms to study neuro-processes. Unitary hydroids have a well-structured and documented CNS (Grimmelikhuijzen and Westfall, 1995; Koizumi, 2016), but evidence of a nervous system in colonial cnidarians is scarce. In his research article, Kosevich reports the presence of a colonial nervous system in the coenosarc of the athecate *Clava multicornis*, the thecate *Dynamena pumila*, and the thecate *Obelia longissima*, all of which are species of colonial hydroids with interconnected zooids. Using confocal scanning laser microscopy ganglion nerve cells, mainly bipolar neurons were visualized. Ultrastructural analysis showed in all studied species (1) the presence of neurons in the coenosarc epidermis, (2) the asymmetry of neuron-muscular synapsis, (3) the settling of neurons and their processes on the mesoglea and the overlapping of the muscle processes over the nerve cells (both in the coenosarc), (4) that some of the neurite's processes run in the mesoglea, and (5) that the density of the nervous system in studied species is different. After analysis, the importance of hydroids as a study model in neuroscience is clear despite the fact that the role of the colonial nervous system is still uncertain.

Although the function of the colonial nervous system in Cnidaria is unclear, new discoveries of possible neuro-communication between host and symbiont are emerging in the phylum Porifera. A good example of this is what Xiang et al. present in their research article. The authors describe a group of monoamine molecules that act as neurotransmitters and/or neuromodulators between the marine sponge *Amphimedon queenslandica* and its holobionts (bacteria symbionts). It is well-known that sponges do not present a nervous system but there is evidence of their integrated paracrine pre-nervous system (Nickel, 2010; Leys and Anderson, 2015). Xiang et al. demonstrates the presence of dopamine and trace amine receptor-like molecules (rhodopsin G-class protein-coupled receptors in sponge larvae). Interestingly, when these larvae are exposed to dopamine agonists or antagonists, the organisms present an evident change in their phototactic swimming behavior, which indicates the possible physiological relationship that exists between sponges and their symbionts.

Coupled with the discoveries of neuroreceptor-type molecules in marine organisms lacking a CNS, in Protostomia clade, the neurosciences have very important animal models, like the giant squid and the sea slug *Aplysia* (Carpenter et al., 1971). New findings are emerging on the functionality of synaptic plasticity, gene-associated synapsis, and neuromodulation in mollusks (Orvis et al., 2022; Zhuo et al., 2022). Bédécarrats et al. analyzed the neuron membrane potential decision during motivated behaviors in the mollusk *Aplysia californica*. They used neuron B63, a decision neuron isolated from the buccal ganglion, that drives radula-biting cycles underlying food-seeking behavior in the sea slug. After synaptic isolation, it was found that B63′s plateau potentials continued after the removal of extracellular Ca^2+^ and were completely suppressed by tetrodotoxin, highlighting the role of voltage-gated Na^+^ channels.

In summary, the present Research Topic has relevant information involving marine organisms from the simplest nervous system to those phylogenetically close to chordates. Having a collection of works about marine invertebrates constitutes an important and invaluable source of information that will certainly have an impact on our current view of the neurosciences.

## Author contributions

TO-B: Writing—review & editing. AO: Writing—review & editing.
